# Conserved aspartic acid 233 and alanine 231 are not required for poliovirus polymerase function in replicons

**DOI:** 10.1186/1743-422X-4-28

**Published:** 2007-03-12

**Authors:** Marion S Freistadt, Karen E Eberle

**Affiliations:** 1 Department of Microbiology, Immunology and Parasitology; Louisiana State University Health Sciences Center, 1901 Perdido St., New Orleans, Louisiana, 70112, USA; 2 Department of Cell and Molecular Biology, 2000 Stern Hall, 6400 Freret St, Tulane University, New Orleans, LA, 70118, USA

## Abstract

Nucleic acid polymerases have similar structures and motifs. The function of an aspartic acid (conserved in all classes of nucleic acid polymerases) in motif A remains poorly understood in RNA-dependent RNA polymerases. We mutated this residue to alanine in a poliovirus replicon. The resulting mutant could still replicate, although at a reduced level. In addition, mutation A231C (also in motif A) yielded high levels of replication. Taken together these results show that poliovirus polymerase conserved residues D233 and A231 are not essential to poliovirus replicon function.

## Background

Poliovirus is a member of the picornaviridae to which rhinovirus and hepatitis A virus also belong. Picornaviruses share many replication strategies including cap-independent translation and a single polyprotein open reading frame. However, central to these viruses are the viral RNA-dependent RNA polymerases (RdRP), responsible for replication of both strands of the viral RNA genome and mRNA transcription. The structures of several RdRP have been solved by X-ray crystallography [1-4]. These polymerases retain the "right-hand" motif of other polymerases, with finger, thumb and palm domains [5]. However, they differ in having a closed active site: it is fully enclosed due to interactions between the fingers and thumb domains.

Canonical motifs were originally recognized by phylogenetic sequence comparisons [6]. The crystal structures have revealed that phylogenetically conserved motifs can be structurally conserved. For RdRPs, these are A, B, C, D and E (diagrammed in [7]). Except for motif E, which is located between the palm and thumb domains, the motifs are clustered in the palm, as suggested earlier [8]. Mutational and biochemical studies, in concert with structural data, have led to hypotheses about the function of these motifs in polymerase function. The function of motif C is best understood, since it contains an absolutely conserved aspartic acid. This aspartic acid is included in Gly-Asp-Asp, which is conserved in most known RdRPs. The function of the aspartic acids is believed to be coordination of the magnesium metal ions, required for activation of the 3'OH of the primer.

The A motif consists of a beta strand, a beta turn and an alpha helix. Although A motifs from the various classes of polymerases differ, the aspartate corresponding to amino acid position 233 in RdRP of poliovirus polymerase (3D^pol^) is conserved among the four classes. The homologous residue (D537) in T7 RNA polymerase is essential for polymerase function and coordinates magnesium, required at the active site [9,10]. In HIV reverse transcriptase the homologous residue (D110) is not essential for RNase H activity, but is for RNA-dependent DNA polymerase function [11] and pyrophosphorolysis [12]. In T7 DNA polymerase the homologous residue Asp-705 binds one metal ion in the crystal structure. However, the function of this conserved residue in RdRP remains poorly understood. Functions of some other conserved residues have been delineated. Mutants in position D238 from 3D^pol ^have a low amount of *in vitro *polymerase activity and render the virus temperature sensitive. Glutamine 710 in motif A in Klenow DdDP provides part of a steric gating mechanism, preventing rNTPs from binding to the polymerase [13].

Alanine 231 is conserved as well, in that the position is valine or glycine among the enteroviruses) [14] and hydrophobic or small residues (alanine, glycine, valine or serine) among DNA polymerases [15].

The functions of motif B and D are not known; however, each forms an alpha helix that may be responsible for proper positioning of the other motifs.

## Results

**Figure 1:**
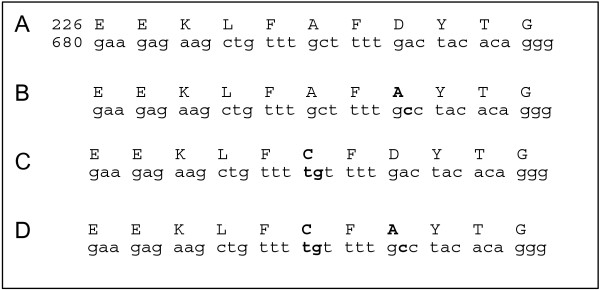
**Location of mutations**. Mutated residues are shown in bold type. Row A presents the wild type amino acid and nucleic acid sequence for part of motif A, starting with glutamic acid at 226 and G at nucleotide position 680 in poliovirus 3D^pol^. Row B shows the mutation that was inserted to construct the mutant D233A. Row C shows the mutation A231C. Row D shows the sequence of the double mutant A231C/D233A.

To determine the importance of residues A231 and D233 of 3D^pol^, we constructed 3 mutant replicons: D233A, A231C and the double mutant A231C/D233A (Figure 1). The site and amino acid mutants we chose to study were obtained during a ribavirin resistance screen (data not shown). Moreover, we wished to determine the function of the conserved D233.

Poliovirus luciferase replicons (kindly provided by R. Andino) consist of a luciferase reporter gene (either firefly or renilla) substituted, in frame, for the capsid region [16]. The renilla luciferase replicon was mutagenized 3 separate times for this study and the firefly replicon once. For mutagenesis, we used the Stratagene Quick Change Kit II. Double stranded DNA encoding the replicon along with the mutagenic primer pair was used as template and primers for pfu Ultra high fidelity enzyme. The mutagenic oligonucleotides consisted of a sense strand of 32–45 bases flanking the mutations and the complimentary oligonucleotide.

For the mutagenesis reaction, target DNA and the mutagenic primers were thermocycled. In this strategy, both strands of the target are synthesized. The first cycle was 95°C for 30 sec. The following 16 cycles were 95°C 30 sec, 55°C 1 min and 68°C for 9 1/2 min (1 minute/kb). The products were then treat with Dpn I for 1 hr at 37°C to remove the unmutagenized bacterial DNA. The reaction products were then transformed into XLI Blue supercompetent cells and plated in the presence of X-gal and IPTG. A mutagenized β-galactosidase (Whitescript) was used as a positive control for mutagenesis. DNA from resulting colonies was sequenced at the LSUHSC sequencing core and proper mutant sequence confirmed. Appropriate plasmids were grown and isolated using qiagen kits.

For introduction of poliovirus replicons into cells, RNA is required. The replicons have a built-in T7 promoter to produce RNA *in vitro*. For RNA transcription, DNA samples were treated with RNase-free conditions, including using DEPC-treated water. Plasmid DNA was first treated with proteinase K in 0.5% SDS at 37°C for 30 min. Following the incubation, the DNA was extracted three times phenol/chloroform and once with chloroform and ethanol precipitated. Then DNA was linearized with Pvu I or Mlu I. After ethanol precipitation, samples were transcribed, using the Megascript T7 kit (Ambion). After adding anti-RNase, the DNA was transcribed for 3 hours at 37°C. Following transcription, the samples were subjected to DNase treatment for 15 min at 37°C and Megaclear columns (Ambion). Samples were inspected on agarose gel electrophoresis for intactness.

To assess the mutants' function, *in vitro *transcribed RNA was electroporated into Hela cells. Hela cells were first trypsinized, washed with PBS and resuspended at 2 × 10^7 ^cells/ml. 2 × 10^6 ^Hela cells were used per transfection. 2 μg of RNA per 10^6 ^cells was added and the samples were incubated on ice for 15 min. The cells and RNA were then aliquotted into 0.2 mm electroporation cuvettes and electroporated at 120 V for 1 pulse and 50 milliseconds in an Electro Square Porator ECM830. Samples were immediately put in warm DMEM + 10% FCS, Pen/Strep. One sample was taken for "time zero" and the remaining cells were split into dishes.

Upon electroporation, the replicons undergo a single round of replication. Because the luciferase gene is in frame with the genome, levels of luciferase reflect the levels of poliovirus replication. Following electroporation, timepoint samples were assessed for luminescence. Cells from each timepoint were scraped, centrifuged and resuspended in 500 μl ProMega "Passive Lysis Buffer" or "Renilla Lysis buffer." After a 15 min room temperature incubation with gentle rocking, samples were centrifuged. Supernatants were collected and stored at -70°C until analysis. A Sealite Sciences luminometer was used to analyze the samples for 10 sec. A renilla and a firefly luciferase assay system from Promega were used to provide the luminescent substrates and diluents. Renilla (Chemicon) and firefly (Promega) positive controls were used.

**Figure 2:**
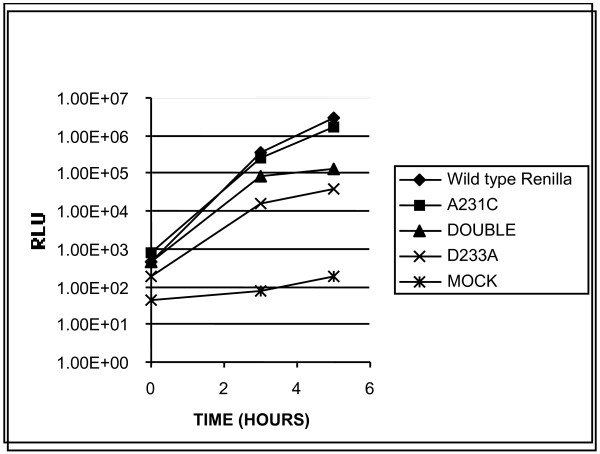
**Luminescence of mutant replicons**. RNA from wild type or mutant renilla luciferase replicons was electroporated into Hela cells and assessed for luminescence at the indicated timepoints. Diamond: wild type replicon; square: A231C; triangle: double mutant A231/D233A; X: D233A, "star"; mock electroporation.

We found that the A231C mutant was almost as active as wild type (Figure 2). The D233A mutant is about two orders of magnitude less active than wild type, but still two orders of magnitude above the "mock" sample. The double mutant is very similar to D233A, suggesting that its defect is primarily due to D233A, rather than A231C. Similar results were obtained with D233A in the firefly luciferase replicon.

Therefore, we conclude that A231 is not required for poliovirus replicon replication. D233 is important for replicon function, but it is not essential. Whether this is true for other RdRPs or polymerases in general, remains to be seen. In 3D^pol^, these two residues are positioned on the same side of the beta strand, pointing slightly away from the heart of the active site. It is noteworthy that in the poliovirus A motif, there is another aspartic acid, at position 238, which points more directly towards the catalytic aspartic acids, D328 and D329 in motif C. It is possible that D238 participates in the hypothesized magnesium coordination in conjunction with D238 and D239. We cannot eliminate the possibility that this finding is peculiar to replicons and may not apply to virus. However, other virus replicon systems have been successfully used to identify functions that apply to virus [17,18].

## Competing interests

The author(s) declare that they have no competing interests.

## References

[B1] Ng KK, Cherney MM, Vázquez AL, Machín A, Alonso JMM, Parra F, James MNG (2002). Crystal structures of active and inactive conformations of a caliciviral RNA-dependent RNA polymerase. J Biol Chem.

[B2] Thompson AA, OB Peersen (2004). Structural basis for proteolysis-dependent activation of the poliovirus RNA-dependent RNA polymerase. Embo J.

[B3] Ago H, Adachi T, Yoshida A, Yamamoto M, Habuka N, Yatsunami K, Miyano M (1999). Crystal structure of the RNA-dependent RNA polymerase of hepatitis C virus. Structure Fold Des.

[B4] Bressanelli S, Tomei L, Rey FA, De Francesco R (2002). Structural analysis of the hepatitis C virus RNA polymerase in complex with ribonucleotides. J Virol.

[B5] Ollis DL, Kline C, Steitz TA (1985). Structure of large fragment of Escherichia coli DNA polymerase I complexed with dTMP. Nature.

[B6] Koonin EV (1991). The phylogeny of RNA-dependent RNA polymerases of positive-strand RNA viruses. J Gen Virol.

[B7] Hansen JL, Long AM, Schultz SC (1997). Structure of the RNA-dependent RNA polymerase of poliovirus. Structure.

[B8] Poch O, Sauvaget I, Delarue M, Tordo N (1989). Identification of four conserved motifs among the RNA-dependent polymerase encoding elements. Embo J.

[B9] Osumi-Davis PA, de Aguilera MC, Woody RW, Woody AY (1992). Asp537, Asp812 are essential and Lys631, His811 are catalytically significant in bacteriophage T7 RNA polymerase activity. J Mol Biol.

[B10] Woody AY, Eaton SS, Osumi-Davis PA, Woody RW (1996). Asp537 and Asp812 in bacteriophage T7 RNA polymerase as metal ion-binding sites studied by EPR, flow-dialysis, and transcription. Biochemistry.

[B11] Boyer PL, Ferris AL, Hughes SH (1992). Cassette mutagenesis of the reverse transcriptase of human immunodeficiency virus type 1. J Virol.

[B12] Kaushik N, Rege N, Yadav PN, Sarafianos SG, Modak MJ, Pandey VN (1996). Biochemical analysis of catalytically crucial aspartate mutants of human immunodeficiency virus type 1 reverse transcriptase. Biochemistry.

[B13] Astatke M, Ng K, Grindley ND, Joyce CM (1998). A single side chain prevents Escherichia coli DNA polymerase I (Klenow fragment) from incorporating ribonucleotides. Proc Natl Acad Sci USA.

[B14] Picornavirus sequence alignments. http://www.iah.bbsrc.ac.uk/virus/picornaviridae/SequenceDatabase/alignments/alignmts.htm.

[B15] Delarue M, Poch O, Tordo N, Moras D, Argos P (1990). An attempt to unify the structure of polymerases. Protein Eng.

[B16] Gohara DW, Crotty S, Arnold JJ, Yoder JD, Andino R, Cameron CE (2000). Poliovirus RNA-dependent RNA polymerase (3Dpol): structural, biochemical, and biological analysis of conserved structural motifs A and B. J Biol Chem.

[B17] Hinzman EE, Barr JN, Wertz GW (2002). Identification of an upstream sequence element required for vesicular stomatitis virus mRNA transcription. J Virol.

[B18] Chen L, Gui C, Luo X, Yang Q, Gunther S, Scandella E, Drosten C, Bai D, He X, Ludewig B, Chen J, Luo H, Yang Y, Yang Y, Zou J, Thiel V, Chen K, Shen J, Shen X, Jiang H (2005). Cinanserin is an inhibitor of the 3C-like proteinase of severe acute respiratory syndrome coronavirus and strongly reduces virus replication in vitro. J Virol.

